# Analysis of Genetic Diversity in Indian Isolates of *Rhipicephalus microplus* Based on *Bm86* Gene Sequence

**DOI:** 10.3390/vaccines9030194

**Published:** 2021-02-26

**Authors:** Balasamudram Chandrasekhar Parthasarathi, Binod Kumar, Gaurav Nagar, Haranahally Vasanthachar Manjunathachar, José de la Fuente, Srikant Ghosh

**Affiliations:** 1 Entomology Laboratory, Division of Parasitology, ICAR-Indian Veterinary Research Institute, Izatnagar 243122, India; parthb763@gmail.com (B.C.P.); gauravn19@gmail.com (G.N.); 2 Department of Veterinary Parasitology, College of Veterinary Science and AH, Junagadh Agricultural University, Junagadh 362001, India; drkumarbinod@gmail.com; 3 ICMR-National Institute of Research on Tribal Health, Nagpur Road, PO. Garha, Jabalpur 482003, India; manjunathachar632@gmail.com; 4 SaBio, Instituto de Investigación en Recursos Cinegéticos, IREC (CSIC-UCLM-JCCM), Ronda de Toledo s/n, 13005 Ciudad Real, Spain; jose_delafuente@yahoo.com; 5 Center for Veterinary Health Sciences, Department of Veterinary Pathobiology, Oklahoma State University, Stillwater, OK 74078, USA

**Keywords:** tick, *Rhipicephalus microplus*, *Bm86* gene, diversity, in silico analysis, tick control, cattle

## Abstract

The control of cattle tick, *Rhipicephalus microplus,* is focused on repeated use of acaricides. However, due to growing acaricide resistance and residues problem, immunization of animals along with limited use of effective acaricides is considered a suitable option for the control of tick infestations. To date, more than fifty vaccine candidates have been identified and tested worldwide, but two vaccines were developed using the extensively studied candidate, *Bm86*. The main reason for limited vaccine commercialization in other countries is genetic diversity in the *Bm86* gene leading to considerable variation in vaccine efficacy. India, with 193.46 million cattle population distributed in 28 states and 9 union territories, is suffering from multiple tick infestation dominated by *R. microplus*. As *R. microplus* has developed multi-acaricide resistance, an efficacious vaccine may provide a sustainable intervention for tick control. Preliminary experiments revealed that the presently available commercial vaccine based on the *BM86* gene is not efficacious against Indian strain. In concert with the principle of reverse vaccinology, genetic polymorphism of the *Bm86* gene within Indian isolates of *R. microplus* was studied. A 578 bp conserved nucleotide sequences of *Bm86* from 65 *R. microplus* isolates collected from 9 Indian states was sequenced and revealed 95.6–99.8% and 93.2–99.5% identity in nucleotides and amino acids sequences, respectively. The identities of nucleotides and deduced amino acids were 94.7–99.8% and 91.8–99.5%, respectively, between full-length sequence (orf) of the *Bm86* gene of IVRI-I strain and published sequences of vaccine strains. Six nucleotides deletion were observed in Indian *Bm86* sequences. Four B-cell epitopes (D519-K554, H563-Q587, C598-T606, T609-K623), which are present in the conserved region of the IVRI-I *Bm86* sequence, were selected. The results confirm that the use of available commercial *Bm86* vaccines is not a suitable option against Indian isolates of *R. microplus*. A country-specific multi-epitope *Bm86* vaccine consisting of four specific B-cell epitopes along with candidate molecules, subolesin and tropomyosin in chimeric/co-immunization format may provide a sustainable option for implementation in an integrated tick management system.

## 1. Introduction

India houses the largest cattle population (193.46 million) in the world [1] and is also the highest producer of milk [2]. However, the per capita productivity is low due to multiple reasons. Among the various reasons, tick infestation is an important contributor to the low-level of animal productivity. Among the 109 species of ticks reported from India, *Rhipicephalus microplus* is a widely distributed species that infests livestock, wildlife, and zoo animals and also causes significant losses to cattle production [3]. This species inhabits India, South East Asia, Central and South America, northern and eastern Australia, eastern and southern Africa, Madagascar, the Mascarene Islands, New Caledonia, and French Polynesia [4,5]. Besides causing a significant reduction in weight gain and milk production, *R. microplus* also transmits *Babesia bigemina, B. bovis, Anaplasmamarginale*in the Indian subcontinent [6]. As per the United Nations Food and Agriculture Organization (FAO) report, 80% of the world’s cattle population is exposed to tick infestation and has an estimated impact of US$7.30/head/year [7]. In India, the cost of controlling ticks and tick-borne diseases (TTBDs) has been estimated at US$498.7 million/annum [8].

The most widely adopted method for tick control is the repeated use of different classes of acaricides. However, indiscriminate use of chemical acaricides “on” and “off” the hosts has led to the emergence and establishment of acaricide-resistant tick populations throughout tropical and subtropical regions of the world [9,10,11,12], including India [13,14]. Besides adding to environmental pollution, the acaricide residues also contaminate milk and meat products [15,16,17,18,19].

Initial studies using native *Bm86* as an immunogen showed significant efficacy against heterologous tick species and later, TickGARD (Hoechst Animal Health; Australia), TickGARD^PLUS^ (Intervet Australia) in Australia, Gavac^TM^ (Heber Biotec; Havana, Cuba) in Latin American countries and BovimuneIxovac (BOVIMUNE IXOVAC) in Mexico was developed and commercialized. The efficacy and benefits of using the anti-tick vaccine as a component of integrated tick management are well established [20,21,22]. The *Bm86* based commercial vaccine provided significant efficacy against some tick strains [23,24,25], and a reduction of the incidence of bovine babesiosis has already been reported [26]. However, the commercial vaccines have shown variable efficacy of 0 to 91% in different geographical areas [20,27,28,29,30,31,32,33,34,35,36,37,38,39,40,41,42], and this has been considered as one of the impediments of commercialization of the vaccine in wide geographical areas. One of the many reasons for the variable efficacy of a recombinant protein-based vaccine is the variability of the *Bm86* amino acid sequence between reference strains used to produce the recombinant vaccines and the field strains [43]. A variation greater than 2.8% in the amino acid sequence of the protein expressed would be sufficient to confer variable efficacy [27]. Partial protection in earlier pen trials [44] with the Cuban r*Bm86* vaccine, Gavac in India indicated variation in the Indian *Bm86* gene sequence. However, investigations have not been conducted to identify the level of diversity in the *Bm86* gene within the Indian strains of *R. microplus* and how this compares to the globally available strains. This information is crucial before exploring the possibility of using the *Bm86* gene for the development of an effective vaccine against Indian cattle tick. Thus, mapping of the *Bm86* variability in strains of interest and prediction of B-cell epitope sequences between Indian and several previously characterized strains, including one commercial tick strain, is targeted in the present study as a guide for the development of effective *Bm86*-based vaccines for India and other countries.

## 2. Materials and Methods

Workflow of the current study mentioned in Supplementary Figure S1.

### 2.1. Tick Samples

The *R. microplus* IVRI-I strain (registration No. NBAII/IVRI/BM/1/1998) was maintained in the Entomology laboratory, Division of Parasitology, ICAR-Indian Veterinary Research Institute, was used as the reference sample. The reference ticks (*N* = 6) (generation 54) were used for the generation of full-length *Bm86* gene sequence. For the *Bm86* gene sequence diversity study, male and female *R. microplus* were collected from cross-bred (*Bos taurus* × *B. indicus*), native Indian breeds of cattle and from buffaloes of 65 districts across India (Supplementary Table S1). The tick isolates were collected between January 2018 to December 2019.

### 2.2. Study Area

Nine states belonging to different agro-climatic zones were selected for sample collection (Supplementary Figure S2). The number of districts from each state was selected based on cattle population [1], tick infestation level and incidence of tick-borne diseases (TBDs). From each district, a pooled sample of about 100–150 ticks were collected following a stratified random sampling procedure and was designated as an isolate. The collected tick samples were cleaned, morphologically identified as *R. microplus* using standard key [30] and stored at −80 °C.

### 2.3. RNA Isolation and cDNA Synthesis

Three engorged female ticks were randomly picked from each field isolate and reference tick strain (IVRI-I), weighed and stored at −80 °C. The ticks were triturated in 2 mL of Trizol^®^ (Thermo Fisher Scientific, Waltham, MA, USA) reagent (1 mL/100 mg tissue). The tick lysate was centrifuged at 10,000 rpm for 5 min; the supernatant was mixed with chloroform (0.2 mL/1 mL of Trizol), incubated at room temperature after vigorous shaking. The aqueous phase was mixed with absolute isopropyl alcohol and centrifuged at 13,000 rpm for 15 min at 4 °C. The supernatant was discarded, and the pellet was washed with 70% ethanol. The RNA pellet was air-dried and allowed to dissolve in DEPC treated water after heating the tube at 55 °C for 15 min. The RNA was stored at −80 °C. The cDNA was synthesized from extracted RNA using a first-strand cDNA synthesis kit (Thermo Fisher Scientific, Waltham, MA, USA) following the manufacturer’s instructions.

### 2.4. Amplification of Full-Length IVRI-I Bm86 (orf) Gene, Cloning and Sequencing

To amplify the *Bm86* cDNA target sequence, a pair of primers were designed using Primer 3 software (https://primer3plus.com/cgi-bin/dev/primer3plus.cgi (accessed on 6 February 2021)). Primers were designed based on the sequence of *R. microplus* reference strain Yeerongpilly (NCBI accession number M29321) as a template. The full-length *Bm86*orf was amplified using the forward (5′-ATG CGT GGC ATC GCT TTA TT-3′; nucleotides 33–52) and reverse (5′-GTT TAG CCC AAC TAT CTT TAT TTG ACA TC-3′; nucleotides 1985–1964) primers. The 25 µL PCR was optimized with the following components: 2.5 µL of 10× DreamTaq green PCR buffer, 1 µL 25 mM MgCl_2_, 0.5 µL of 10 mM dNTPs (Thermo Fisher Scientific, Waltham, MA, USA), 1 µL 50 ng cDNA, 10 µM of each primer, 0.3 µL DreamTaq DNA polymerase (5 U/µL) (Thermo Fisher Scientific, Waltham, MA, USA) and sterile nuclease-free water in sufficient quantity to make up the volume. The PCR (Veriti 96-well thermal cycler, Applied Biosystems, Foster City, CA, USA) condition was: 4 min at 95 °C followed by 35 cycles of denaturing step of 30 s at 95 °C, an annealing step of 40 s at 62.5 °C, an extension step of 2 min at 72 °C and a final extension step of 72 °C for 15 min. The band representing the 1953 bp *Bm86* cDNA amplicon was excised and purified using a QIAquick gel extraction kit (Qiagen, Hilden, Germany). The purified PCR products were cloned in a pTZ57R/T cloning vector (Thermo Fisher Scientific, Waltham, MA, USA). Preparation of competent cells and transformation was carried out using Transform Aid^TM^Bacterial transformation kit (Thermo Fisher Scientific, Waltham, MA, USA) following manufacturer’s protocol. Identification of positive clones was based on blue-white colony screening and colony PCR. Subcultures of positive clones were outsourced for double-stranded sequencing to the Department of Biochemistry, Delhi University, New Delhi. Generated sequences were analyzed, annotated and submitted to GenBank (NCBI, Bethesda, MD, USA).

### 2.5. Amplification of the Bm86 Conserved Sequence

In order to design an effective *Bm86*-based vaccine, a conserved sequence of 192 amino acids (from 438th to 629th a.a.) was identified after multiple sequence alignment with published sequences. Primers were self-designed from the *Bm86* Indian sequence (Accession No. MK728951) as forward (5′-TGC GAC AGT CTG CTC AAG AAT-3′; nucleotides 1306–1326) and reverse (5′-GCT GCA GCA CTT GAC TTT CCA-3′; nucleotides 1883–1863). The PCR conditions were optimized as; 4 min at 94 °C followed by 35 cycles of a denaturing step of 30 s at 94 °C, an annealing step of 30 s at 52 °C, an extension step of 1 min at 72 °C and a final extension step of 72 °C for 15 min. A total of 195 PCR were performed (65 × 3: three reactions per cDNA sample). The band representing 578 bp *Bm86* cDNA amplicon was excised, purified and cloned as mentioned above. The positive clones (five in each isolate) were outsourced for single-stranded sequencing. Each generated sequence was analyzed, annotated and submitted to GenBank.

### 2.6. Phylogenetic Analysis

Nucleotide and amino acid sequences were aligned with Clustal*W*, BioEdit software (Version 7.0.5.3) (BioEdit Limited, Manchester, England), and phylogenetic analysis was performed using neighbor-joining and maximum-likelihood methods and based on the P-distance and Jones–Taylor–Thornton (JTT) model. Phylogenetic and molecular evolutionary analyses were conducted using MEGA X [31]. Bootstrap analysis was conducted using 1000 replicates to assess the reliability of inferred tree topologies.

### 2.7. In Silico Prediction of Linear B-Cell Epitopes on IVRI-IBm86 Protein

To predict linear B-cell epitopes, a combination of two prediction algorithms were used: Bepipred 2.0 (http://tools.immuneepitope.org/bcell/) and VaxiJen (http://www.ddgpharmfac.net/vaxijen/VaxiJen/VaxiJen.html).

First, the prediction of linear B-cell epitopes was carried out using the IEDB web server Bepipred 2.0 (National Institute of Allergy and Infectious Diseases, Bethesda, MD, USA). For each FASTA input sequence, a prediction score for each amino acid was obtained. To determine potential B-cell linear epitopes, we utilized the recommended cutoff of 0.5 [45], where an average score of at least nine consecutive amino acids were used for determining the cutoff. Sequences with a Bepipred score above 0.5 were considered as potential linear B-cell epitopes and analyzed by VaxiJen, the first server used for alignment-independent prediction of protective antigens. It was developed to allow antigens classification based on the physicochemical properties of proteins without recourse to sequence alignment. Bacterial, viral, parasite and tumor protein datasets were used to derive models for the prediction of whole protein antigenicity with prediction accuracy from 70% to 89% [45,46]. To evaluate the antigenicity of predicted epitopes, we utilized the default cutoff (0.5), suggested to parasite antigens. Therefore, sequences with a Bepipred score above 0.5 and a VaxiJen score above 0.5 were considered potential linear B-cell epitopes and evaluated for specificity.

### 2.8. Evaluation of Degree of Conservation of Linear B-Cell Epitopes

Sequences identified as potential linear B-cell epitopes were aligned to amino acid sequences of IVRI-I *Bm86* for comparison with reference sequences, Yeerongpilly(Accession No: M29321), Camcord (Cuba), USA (Hidalgo) (Accession No: HQ014395), USA (Zapta 1) (Accession No: HQ014393), Brazil (Campo Grande) (Accession No: EU352677), Thailand (M1) (Accession No: KJ995883), Thailand (M2) (Accession No: KJ995884), China (XJNJ) (Accession No: MH165269), Mexico (Accession No: FJ456928), Mozambique (Accession No: FJ809946) using BioEdit sequence alignments editor, Version 7.0.9.0.

**Accession numbers generated:** A total of 66 sequences were submitted to NCBI (65 *Bm86* conserved sequences and one full-length *Bm86* sequence)

MK728951, MN088493, MN095773, MN095774, MN095775, MN095776, MN095777, MN095778, MN095779, MN095780, MN095781, MN095782, MN095783, MN095784, MN095785, MN095786, MN095787, MT503269, MN115793, MN115794, MN115795, MN115796, MN115798, MN115797, MN319498, MN319497, MT503270, MN585696, MN585697, MN585698, MN585699, MN585700, MN585701, MN585702, MN585703, MN585704, MN585706, MT503271, MN585687, MN585701, MN585689, MN585690, MN585691, MN585692, MN585693, MN585694, MN585695, MT503262, MT503263, MT503264, MT503265, MT503266, MT503267, MT503268, MT503272, MT503273, MT503274, MT503275, MT503276, MT503277, MT503278, MT503279, MT503280, MT503281, MT503282, MN585705.

## 3. Results

### 3.1. Sequence Analysis

The full-length and partial targeted sequences of the *Bm86* gene were amplified as 1953 bp and 578 bp, respectively, without any nonspecific reactions (Supplementary Figure S3). One of the objectives of the study was to measure the level of polymorphism between Indian (IVRI-I) *Bm86* gene with worldwide published full-length *Bm86* gene sequences. The amino acid sequence identity matrix (Table 1) revealed that the Indian (IVRI-I) *Bm86* protein has 93.2% homology (6.76% polymorphism) and 92.7% (7.22% polymorphism) with the Yeerongpilly (TickGARD^TM^) and Camcord (Cuba) (GAVAC) vaccine strains, respectively. The multiple sequence alignment (MSA) analysis showed (Supplementary Figure S4) that the specific amino acids of IVRI-I *Bm86* differs from the Yeerongpilly vaccine strain at 44 loci, including 42 substitutions and 2 deletions (186, 187) and also differs from Camcord (Cuba) vaccine strain at 44 loci (44 substitutions) (Supplementary Table S2).

### 3.2. Phylogenetic Analysis

**Figure 1:**
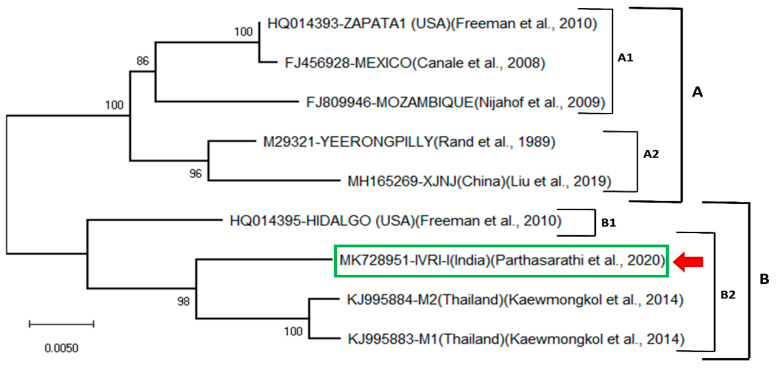
Neighbor-joining phylogenetic tree of the *Bm86* mRNA sequence from *R. microplus* IVRI-I strain. Percentage bootstrap support from 1000 pseudo-replicates is indicated at the left of the supported nodes, based on evolutionary distances calculated with the P-distance model.

Figure 1 shows the phylogenetic tree of the IVRI-I nucleotide sequence with 9 other sequences from different isolates using the neighbor-joining method based on the P-distance model. Two main clades, A and B, were formed. Clade A consists of two subclades (A1, A2). The A1 is formed by three sequences of Zapata 1 (USA), Mexico and Mozambique. Subclade A2 corresponds to two sequences of Yeerongpilly and XJNJ (China). Clade B is formed by 4 sequences. It forms two subclades: one small B1 and a large B2. The subclade B1 is formed by the IVRI-I sequence, and the subclade B2 is mainly represented by two sequences of Thailand. It is observed that the IVRI-I sequence is closely related to two Thailand isolates (M1 and M2) and Hidalgo isolate (USA) rather than the Yeerongpilly, China and other strains. Whereas, Yeerongpilly vaccine strain is closer to the Chinese isolate and forms a separate sister group and more distant to Indian and Thailand isolates (Figure 1). The nucleotide sequences identity matrix revealed that the IVRI-I isolate had 95% homology with that of the Cuban and Australian isolates, respectively (Table 2).

**Figure 2:**
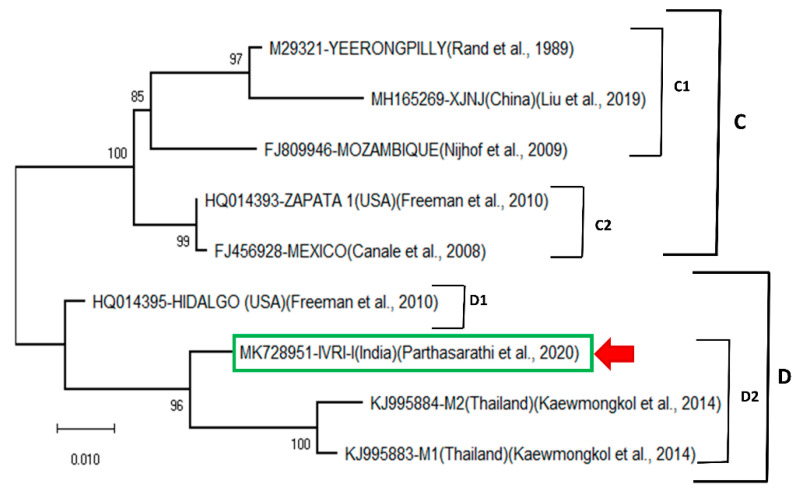
Maximum-likelihood tree of the *Bm86* amino acid sequences from *R. microplus* IVRI-I strain. Percentage bootstrap support from 1000 pseudo-replicates is indicated at the left of the supported nodes, based on evolutionary distances calculated with the Jones–Taylor–Thornton (JTT) model.

When the same *Bm86* sequences of the deduced amino acid were compared using a maximum-likelihood tree based on Jones–Taylor–Thornton (JTT) model (Figure 2), two clades (C, D) are formed. The clade C consists of two subclades (C1, C2). The C1 subclade is formed by three sequences of Yeerongpilly, XJNJ (China) and Mozambique. The subclade C2 consists of two sequences of Zapata1 (USA) and Mexico. The clade D is formed by 4 sequences and divided into two subclades (D1, D2). The subclade D1 is formed by Hidalgo (USA) sequence, and IVRI-I and Thailand M1, M2 are together formed subclade D2. The IVRI-I strain was closely related to two Thailand isolates (M1, M2), while Hidalgo isolates of the USA formed a single cluster (Figure 2). The Chinese isolates were closely related to the Yeerongpilly reference sequence and formed a single clade. Here again, except for, Hidalgo isolate, the remaining *Bm86* sequences from USA were arranged in a single clade and clustered with the China-Yeerongpilly clade. The *Bm86* amino acid identity matrix (Table 1) revealed that the IVRI -I *Bm86* has the sequence identity of 93.2% and 92.7% with the Yeerongpilly and Camcord (Cuba) vaccine strains, respectively, and the highest sequence identity (96.6%) was observed with the Thailand M2 isolate.

**Figure 3:**
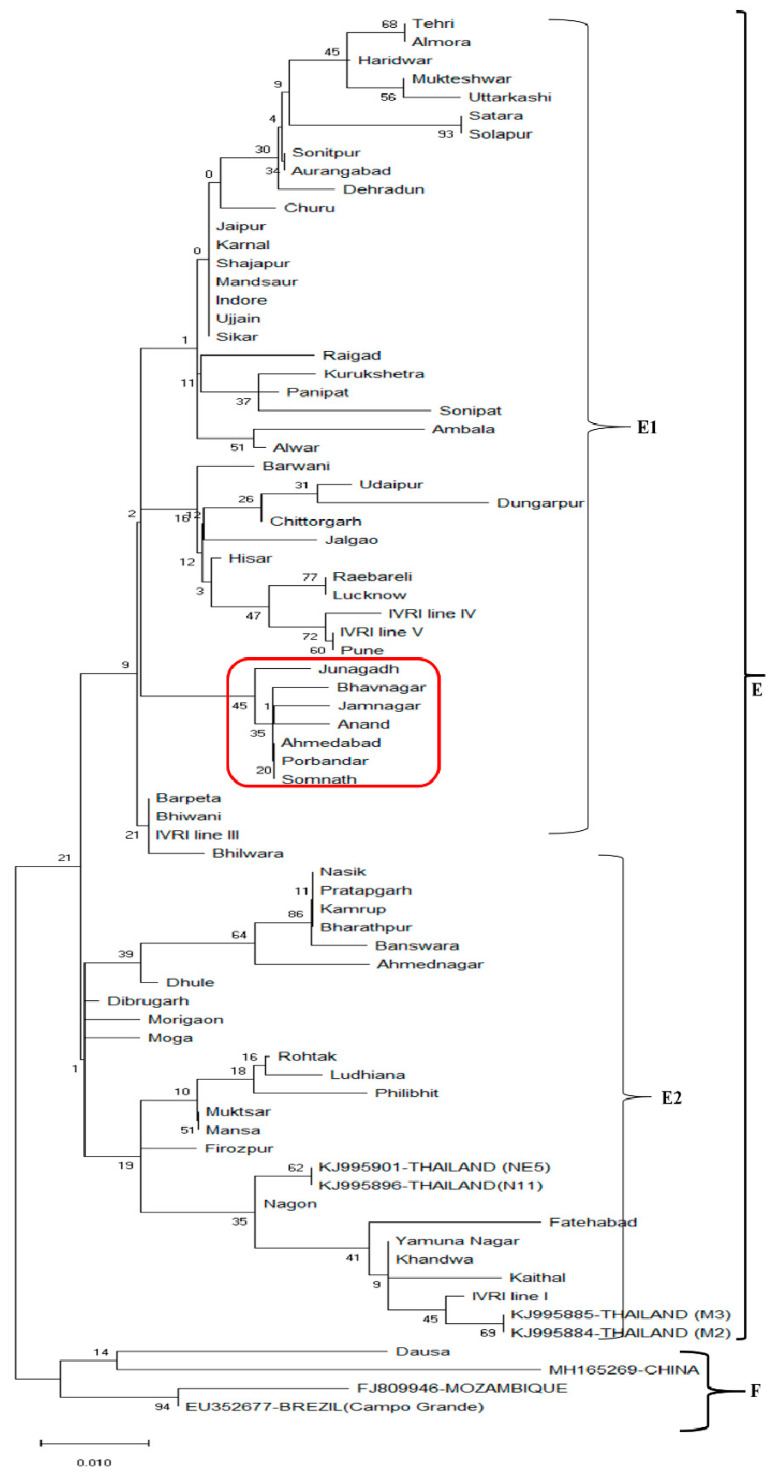
Maximum-likelihood tree of the conserved *Bm86* amino acid sequences of *R. microplus* isolates in India, Thailand, China, China, Mozambique and Brazil (Campo Grande). Percentage bootstrap support from 1000 pseudo-replicates is indicated at the left of the supported node, which is based on evolutionary distances calculated with the Jones–Taylor–Thornton (JTT) model.

The other objective of the present study was to assess the level of polymorphism in conserved *Bm86* sequences of different Indian and in other isolates. Analysis of partial sequences of the *Bm86* generated from different Indian isolates revealed 95.6% to 99.8% and 93.2% to 99.5% identity in nucleotides and amino acids sequences, respectively. Phylogenetic comparison of the sixty-five Indian sequences with the published sequences from Thailand, China, Mozambique and Brazil (Campo Grande) formed two distinct clades (E and F). The clade F is formed by the conserved sequences of China, Mozambique, Brazil (Campo Grande) and the Dausa sequence of India. Clade E consists of the highest number of conserved sequences; however, most of them do not form any identifiable subclades due to high diversity. The cade E is subdivided into subclade E1 and E2. The subclade E1 is formed by forty-three conserved sequences of Indian states. Interestingly, the Gujarat sequences were arranged in a group (red color box) (Figure 3). The subclade E2 is formed by twenty-two conserved sequences of different Indian isolates and four *Bm86* sequences of Thailand isolates. On comparing the 578 bp sequence of sixty-five Indian isolates with a sequence of IVRI-I, few specific amino acid changes/ mutations were observed. The conserved *Bm86* Indian isolates showed a minimum (1) to maximum (11) different amino acid substitutions/mutations (Supplementary Table S3). Analysis of the state-wise share of total amino acid substitutions revealed that Rajasthan state is contributing a maximum share of (20%) substitutions/mutations and acting as the geographical hot spot of the *Bm86* mutations. The state-wise share of amino acid mutations (geographical hot spots) in conserved *Bm86* is as follows, Rajasthan (20%)> Maharashtra and Haryana (15%)> Madhya Pradesh and Gujarat (12%) > Uttarakhand and Assam (9%)> Uttar Pradesh and Punjab (4%) (Supplementary Figure S5).

### 3.3. In Silico Analysis of IVRI-I Bm86 Protein

The Bepipred 2.0 algorithm (threshold 0.5) predicted seventeen B-cell epitopes in the IVRI-I *Bm86* sequence. Nine epitopes (T18-D45; D97-G129; G177-D224; W280-R311; K319-K501; D519-K554; H563-Q587; C598-T606; T609-K623) were selected after processing of each epitope in the VaxiJen server. The epitopes showing more than 0.5 VaxiJen scores were considered as probable antigenic epitopes and are shown in yellow color (Table 3). The predicted potential B-cell epitopes in IVRI-I *Bm86* protein werecompared with published *Bm86* strains amino acid sequences, and percentage similarity is tabulated (Table 4). The three epitopes *Bm86* (T18-D45), *Bm86* (D519-K554) and *Bm86* (H563-Q587) of IVRI-I *Bm86* are highly similar (100%) to worldwide published reference strain B-cell epitope sequences.The percentage amino acid similarity of IVRI-I *Bm86* and its predicted epitopes compared to eight worldwide published *Bm86* strains amino acid sequences were as follows: Bm86C598-K606, 88.8 to 100% (medium to high); T609-K623, 86.6 to 100% (medium to high); *Bm86* W280-R311, 81.2 to 93.7% (low to medium); *Bm86* G177-D224, 82 to 97.9% (low to medium): Bm86D97-G129, 78.7–96.9% (low, medium, and high) (Table 4).

Nine B-Cell epitopes were predicted in full-length (648 a.a.) IVRI-I *Bm86* sequence. Only four B-cell epitopes of field isolates (D519-K554, H563-Q587, C598-T606, T609-K623) come within the range of conserved sequence (438th to 629th amino acid of IVRI-I *Bm86*), and similarity percentage is tabulated (Supplementary Table S4). The epitopes, D519-K554 and T609-K623, in all the nine isolates showed 100% similarity. The epitope C598-K606 showed medium (91%) (Uttar Pradesh) and high (100%) similarity with the remaining eight field isolates. The epitope, H563-Q587, showed a medium similarity of 88% (Uttarakhand), 92% (Madhya Pradesh and Punjab), and the remaining 5 field isolates showed high similarity (100%). The similarity percentage of four epitopes in the conserved sequence was also compared with published reference sequences and tabulated in Table 4. The epitopes, D519-K554 and H563-Q587, in all the eight reference epitope sequences, showed 100% similarity. The epitopes C598-K606 showed medium (88.8%) (China) and high similarity (100%) with the remaining 7 reference epitope sequences. The epitopes T609-K623 showed medium (86.6%) (China) and high similarity (100%) with the remaining 7 reference epitope sequences.

The B-cell epitope wise amino acid substitutions and deletion effect on B-cell epitope antigenicity were correlated with the VaxiJen scores of IVRI-I and vaccine strains, Yeerongpilly and Camcord. The scores are tabulated in Supplementary Table S5. Interestingly,

1. Two deletions in IVRI-I epitopes (G177-D224) showed increased antigenicity to Yeerongpilly and Camcord strains;
2. same VaxiJen scores were observed in four epitopes (T18-D45, D519-K554, C598-T606, T609-K623) of IVRI-I, Yeerongpilly and Camcord strains; the same score may be due to 100% similarity in the epitope sequence;
3. the substitutions/ mutations can increase (G177-D224, W280-R311) or decrease (D97-G129, K319-K501, H563-Q587) the antigenicity (VaxiJen scores) of IVRI-I epitopes when compared to the same epitopes in Yeerongpilly and Camcord strains (Supplementary Table S5).

## 4. Discussion

In India, due to problems associated with tick infestations in animals and the ever-increasing problem of selection and establishment of acaricide-resistant tick populations, the demand for alternative control strategies, including an anti-tick vaccine, is very high. Identification of vaccine targets is key to the success of any vaccine, and genetic homogeneity of the identified candidate antigen(s) is to be assured before further experimentation. Accordingly, the present study was designed to evaluate the variation in the *Bm86* gene sequence of Indian *R. microplus* strains. The sequencing analysis of IVRI-I *Bm86* revealed that the *Bm86* gene is 648 amino acids long with two amino acid mutations. In contrast, most of the other reference sequences are 650 amino acids long. Similarly, amino acid deletions were also seen in the Hidalgo isolate of the USA and Chennai isolate of India.

The phylogenetic analysis of nucleotide and amino acid sequence of IVRI-I *Bm86* with published reference *Bm86* sequences revealed that IVRI-I *Bm86* is evolutionarily closely related to Thailand isolates and distant to commercial vaccine strains (Yeerongpilly, Camcord, Mexico and others). This may be due to the geographical location of Thailand isolates, which are closer than other countries, and these data are in agreement with the previous observations by Kaewmongkol and coworkers’ [29]. The sequence identity matrix analysis showed that the IVRI-I *Bm86* protein has 93.2% homology (6.76% divergence) and 92.7% (7.22% divergence) with the Yeerongpilly (TickGARD^TM^) and Camcord (Cuba) (GAVAC) vaccine strains, respectively. The divergence level of more than 2.8% has been reported as a limiting factor in the variation of efficacy of the *Bm86*-based vaccines [27,29]. The sequence divergence data validates the earlier observation in which 44.5% and 25.1% efficacy against *R. microplus* (IVRI-I strain) and *Hyalomma anatolicum* (IVRI-II strain), respectively, was recorded in a pen trial using commercial Cuban *Bm86* vaccine [44]. The high diversity of IVRI-I *Bm86* and low efficacy of commercial Mexican *Bm86* vaccine in India showed that there is a strong need foran Indian-specific *Bm86* vaccine.

Multiple sequence analysis of 578 bp IVRI-I conserved sequences with 65 Indian field isolates revealed 95.6 to 99.8% and 93.2 to 99.5% identity in nucleotides and amino acids sequences, respectively (Supplementary Table S4).

The analysis of the state-wise total number of substitutions/mutation (presented in pie chart form) (Supplementary Figure S5) revealed that Rajasthan state contributes the maximum share (20%) and Uttar Pradesh and Punjab states contribute the minimum share (4%). India is a highly diversified country in terms of geography, climatic conditions, and cattle breeds. A significant level of polymorphism among Indian *Bm86* may have resulted from the adaptation of the tick species to different climatic conditions and cattle breeds. The *R. microplus* isolates from the various regions have undergone different environmental pressures, and these may have influenced the physiological, morphological and genetic variations among these isolates.

Due to the diversity in full-length IVRI-I *Bm86* gene sequences and in conserved *Bm86* sequence of different field isolates, the development of the *Bm86* antigen-based vaccine using the entire *Bm86* sequence under Indian conditions may not give maximum protection against *R. microplus.* Instead of using a whole antigen vaccine, epitope-based vaccines have advantages, such as safety, specificity, and low production cost. For example, due to its significant efficacy, WHO has approved a multi- epitope-based malaria vaccine (RTS, S (Mosquirix™)) for human use [47]. Accordingly, in the present study, the IVRI-I *Bm86* antigen was screened for B-cell epitopes.

Nine liner B-cell epitopes were identified after screening through Bepipred and VaxiJen servers. Four epitopes (D519-K554, H563-Q587, C598-T606, T609-K623), which are present in the conserved region of the IVRI-I *Bm86* sequence, were selected. The similarity percentage of these epitopes with published *Bm86* reference sequences was in the range from 86.6 to 100%, while with conserved *Bm86* Indian field isolates, it was from 88 to 100%. The analysis of the impact of substitution/mutations in antigenicity/immunogenicity of B-cell epitope based on VaxiJen scores revealed that the amino acid deletion of G177-D224 in IVRI-I *Bm86* epitope increased the epitope antigenicity. The in silico analysis of the impact of deletions and substitutions/mutations on the antigenicity of B-cell epitopes provided an idea of the predicted efficacy of the vaccine.

In the related fields of vaccine research, similar results have been reported. For example, initially, the apical membrane antigen 1 (AMA-1) was proposed as the most suitable subunit vaccine candidate for apicomplexan parasites, including *Eimeria tenella* [33], *E. maxima* [34], and *P. falciparum* [35,36,37]. However, high allelic diversity, with more than 60 polymorphic amino acids, has limited the development of an AMA-1-based *P. falciparum* vaccine [36,37,38,39]. In India, while exploring the possibility of developing vaccines against *E. tenella, P. falciparum* and *Theileria annulata*, high single nucleotide polymorphism (SNP) haplotype diversity in south Indian isolates of *E. tenella* compared to north Indian isolates [40] was noticed. High allelic sequence variation in merozoite surface antigen-1 (MSA-1) between 98 Indian isolates of *P. falciparum* [41], *P. falciparumPfg377* gametocyte gene in 122 field isolates [42] and *Tams1* gene of *T. annulata* parasite [48] were observed and vaccine development work was reoriented accordingly. The results of the present study and the lesson learned from the earlier experiments suggest that the Indian *Bm86* protein sequence is showing high polymorphism. The B-cell epitope analysis and diversity study revealed four India-specific B-cell epitopes (D519-K554, H563-K606, C598-K606, T609-K623), which were common and highly similar in all the isolates collected across the country. The current study also identified six B-cell epitopes (T18-D45, K319-K501, D519-K554, H563-K606, C598-K606, T609-K623), which were common in all the commercial *Bm86* vaccine strains, including the Indian *Bm86* sequence. These B-cell epitopes will be helpful in designing future universal multi-B-cell-epitope-based *Bm86* vaccine. The four India-specific *Bm86* B-cell epitopes along with other tick vaccine molecules viz., subolesin [49,50], tropomyosin [51] in chimeric vaccine/ co-vaccination format may be suitable for *R. microplus* management under Indian conditions.

## 5. Conclusions

A significant level of polymorphism in the full-length *Bm86* gene and 65 conserved *Bm86* sequences were found in *R. microplus* populations collected from 9 states of India. Based on the present sequence diversity study and previous in vivo pen trials, data showed that commercial vaccines based on whole antigen *Bm86* vaccines might not be suitable under Indian conditions. Future studies should be on the diversity study on India-specific *Bm86* B-cell epitope sequences. Sampling sites should include other states that were not part of the current work. Additionally, studies should aim to develop India-specific multi-B-cell epitope-based chimeric/cocktail/co-vaccination strategies using computational technologies.

## Figures and Tables

**Table 1:** Deduced amino acid sequence identity matrix of published full-length *Bm86* sequences with Indian *Bm86* sequence.

	A	B	C	D	E	F	G	H	I	J	K	L
A	ID	100	93.2	94.4	90.1	96.7	97.8	93	93.3	28.6	93.5	90.7
B	100	ID	93.2	94.4	90.1	96.7	97.8	93	93.3	28.6	93.5	90.7
C	93.2	93.2	ID	97	86.9	93.6	92.4	96.3	96.6	28.3	89.3	87.5
D	94.4	94.4	97	ID	88.7	95.5	93.5	94.6	94.9	29.3	91.5	89
E	90.1	90.1	86.9	88.7	ID	92.9	89.5	86.4	86.7	27	93.5	99
F	96.7	96.7	93.6	95.5	92.9	ID	95.8	93.2	93.5	28.6	93.8	93.5
G	97.8	97.8	92.4	93.5	89.5	95.8	ID	91.8	92.1	27.6	92.6	89.8
H	93	93	96.3	94.6	86.4	93.2	91.8	ID	98.9	27.5	88.9	87
I	93.3	93.3	96.6	94.9	86.7	93.5	92.1	98.9	ID	27.5	89.2	87.3
J	28.6	28.6	28.3	29.3	27	28.6	27.6	27.5	27.5	ID	26.1	27.3
K	93.5	93.5	89.3	91.5	93.5	93.8	92.6	88.9	89.2	26.1	ID	93
L	90.7	90.7	87.5	89	99	93.5	89.8	87	87.3	27.3	93	ID

A = Yeerongpilly, B = Camcord (Cuba), C = IVRI-I (India), D = Hidalgo (USA), E = Campo grande (Brazil), F = Zapata 1 (USA), G = XJANJ (China), H = M2 (Thailand), I = M1 (Thailand), J = Chennai (India), K = Mozambique, L = Mexico.

**Table 2:** Sequence identity matrix of published full-length *Bm86* nucleotide sequences with the Indian *Bm86* sequence.

	A	B	C	D	E	F	G	H	I	J	K	L
A	ID	100	95	95.6	91.3	98.1	98.6	95.5	95.5	28.9	94.8	91.7
B	100	ID	95	95.6	91.3	98.1	98.6	95.5	95.5	28.9	94.8	91.7
C	95	95	ID	97.3	88.4	95.1	94.8	97.5	97.5	28.6	91.9	88.7
D	95.6	95.6	97.3	ID	89.6	96.3	95.6	96.4	96.4	29.2	92.7	89.7
E	91.3	91.3	88.4	89.6	ID	93.1	90.6	88.7	88.7	27.5	94.5	99.4
F	98.1	98.1	95.1	96.3	93.1	ID	97.2	95.5	95.5	28.9	95	93.4
G	98.6	98.6	94.8	95.6	90.6	97.2	ID	94.9	94.9	28.3	94	90.8
H	95.5	95.5	97.5	96.4	88.7	95.5	94.9	ID	99.5	28.3	92.2	89
I	95.5	95.5	97.5	96.4	88.7	95.5	94.9	99.5	ID	28.3	92.2	89
J	28.9	28.9	28.6	29.2	27.5	28.9	28.3	28.3	28.3	ID	26.4	27.5
K	94.8	94.8	91.9	92.7	94.5	95	94	92.2	92.2	26.4	ID	94.2
L	91.7	91.7	88.7	89.7	99.4	93.4	90.8	89	89	27.5	94.2	ID

A = Yeerongpilly, B = Camcord (Cuba), C = IVRI-I (India), D = Hidalgo (USA), E = Campo grande (Brazil), F = Zapata 1 (USA), G = XJANJ (China), H = M2 (Thailand), I = M1 (Thailand), J = Chennai (India), K = Mozambique, L = Mexico.

**Table 3:** The list of high scored predicted B cell epitopes of IVRI-I *Bm86* strain using IEDB server and their VaxiJenscores ^a^.

B- Cell Epitope Sequence	Length	IEDB Server Score	VaxiJen Score
18TAESSICSDFGNEFCRNAECEVVPGAE45	28	0.52	**0.795 (Probable ANTIGEN)**
24DNMYFNAAEKQCEYKDTCKTRECS77	24	0.54	Probable NON-ANTIGEN
83QSNP86	4	0.5	Probable NON-ANTIGEN
97DTLTLQCNIKDDYATDCRNSGGTAKLRTDGVIG129	33	0.58	**1.3361 (Probable ANTIGEN)**
136EWGAMNKTTRN146	11	0.52	Probable NON-ANTIGEN
152CLRPDLTCKDLCEKNLLQRDSR173	22	0.55	Probable NON-ANTIGEN
175C175	1	1.0	Probable NON-ANTIGEN
177GWNSPKCSAPADSYCSPGSPKGPDGQCKDACKTKEAG FVCKHGCRSTD224	48	0.54	**0.9020 (Probable ANTIGEN**)
235FTVAEDGITCKSIPYTGGCTVEQKQTCR262	28	0.54	Probable NON-ANTIGEN
280WNQHLVGDKCIGDCVENKCHGEFTDCGVYMNR311	32	0.536	**1.3679 (Probable ANTIGEN)**
319KSRKPGPNVNINECLLNEYYYTVSFTPNISLDSDHCDWYED RVLEAIRTSIGKEVFKVEILNCTQDIKARLIAEKPLSKHVL RKLQACEHPIGEWCMMYPKLLIKKNSATEIEEENLCDSLLK NQEAAYKGQNKCVKVDNLFWFQCADGYTTTYEMTRGRLR RSVCKAGVSCNENEQLECADK501	183	0.57	**0.6052 (Probable ANTIGEN)**
507YEN509	3	0.50	Probable NON-ANTIGEN
511K511	1	1.0	Probable NON-ANTIGEN
519DTKPGEIGCIERTTCNPKEIQECQDKKLECVYKNHK554	36	0.56	**0.8751 (Probable ANTIGEN)**
563HECSREPAKDSCSEEDNGKCQSSGQ587	25	0.55	**1.4514 (Probable ANTIGEN)**
598CKEKSEATT606	9	0.510	**1.4151 (Probable ANTIGEN)**
609TTTTKAKDKDPDPGK623	15	0.58	**0.7273 (Probable ANTIGEN)**

^a^ Bolded epitopes are selected epitopes with more than 0.5 VaxiJen scores.

**Table 4:** Values express the percentage similarity of tick strain IVRI-I *Bm86* and its predicted epitopes among worldwide published *Bm86* strains amino acid sequences. The levels of amino acid similarity were classified as low (75–85%; green cells), medium (85–95%; yellow cells) and high (95–100%; red cells).

Predicted IVRI-I *Bm86* B-cell Epitopes	Yeerongpilly	Thailand M1	Thailand M2	Thailand S1	USA (Hidalgo)	USA (Zapta 1)	USA (Starr 2)	China
*Bm86*	93%	96.4%	96.1%	96.3%	97.0%	93.5%	93.3%	92.3%
*Bm86*(T18-D45)	100%	100%	100%	100%	100%	100%	100%	100%
*Bm86*(D97-G129)	84.8%	96.9%	96.9%	93.9%	84.8%	81.8%	81.8%	78.7%
*Bm86*(G177-D224)	82.0%	84%	84%	84%	97.9%	84%	84%	82%
*Bm86*(W280-R311)	81.2%	93.7%	90.6%	93.7%	93.7%	81.2%	81.2%	81.2%
*Bm86*(K319-K501)	97.8%	97.2%	96.7%	97.8%	98.9%	97.2%	96.7%	98.3%
*Bm86*(D519-K554)	100%	100%	100%	100%	100%	100%	100%	100%
*Bm86*(H563-Q587)	100%	100%	100%	100%	100%	96%	100%	100%
*Bm86*(C598-K606)	100%	100%	100%	100%	100%	100%	100%	88.8%
*Bm86*(T609-K623)	100%	100%	100%	100%	100%	100%	100%	86.6%

## Data Availability

The data sets used and/or analyzed during the present study are available from the corresponding authors on reasonable request.

## Supplementary Materials

The following are available online at https://www.mdpi.com/2076-393X/9/3/194/s1, Figure S1: Flowchart of study methodology. Figure S2: An outline map showing district wise sample collection sites. Figure S3: PCR amplification of full-length (1947 bp) and 578bp fragment of conserved *Bm86* gene sequence of IVRI-I (lane M: 100bp plus DNA ladder (Thermo Scientific, USA); lane 1:1947 bp PCR product; lane N: 100 bp DNA Ladder (Gold Biotechnology, USA); lane A: 578 bp PCR product). Figure S4: Multiple align sequence analysis of full-length IVRI-I *Bm86* gene with published vaccine strains; green box depicting the amino acid deletion in Indian *Bm86* sequence; red box showing the *Bm86* conservation sequence. Figure S5: Pie chart representing the percentage of total amino acid share of each state (each state conserved *Bm86* compared to IVRI-I *Bm86* conserved sequence). Table S1: Location of Rhipicephalus microplus engorged female tick samples collected across India. Table S2: Specific amino acid substitutions/mutations of full-length Indian (IVRI-I) *Bm86* gene with respect to commercial vaccine strains. Table S3: Specific amino acid substitutions/mutations in different Indian isolates conservation sequences with respect to Indian (IVRI-I) *Bm86* conservation sequence.Table S4: The percentage similarity of conserved IVRI-I *Bm86* B-cell epitopes with Indian *Bm86* conserved field isolates B-cell epitopes. The levels of amino acid similarity were classified as low (75–85%; green cells), medium (85–95%; yellow cells) and high (95–100%; red cells). Table S5: Table S5: The impact of mutations on IVRI-I *Bm86* B-cell epitope antigenicity with respect to the *Bm86* vaccine strains (based on VaxiJen score analysis)
