# Supplementary material for: Analysis of Genetic Diversity in Indian Isolates of Rhipicephalus microplus Based on Bm86 Gene Sequence
Source: Vaccines (Basel). 2021 Feb 26;9(3):194. doi: 10.3390/vaccines9030194 (PMC7996562; doi:10.3390/vaccines9030194)
Supplement: Supplementary file 1 [file vaccines-09-00194-s001.pdf]

Supplementary Materials:

Supplementary figures:

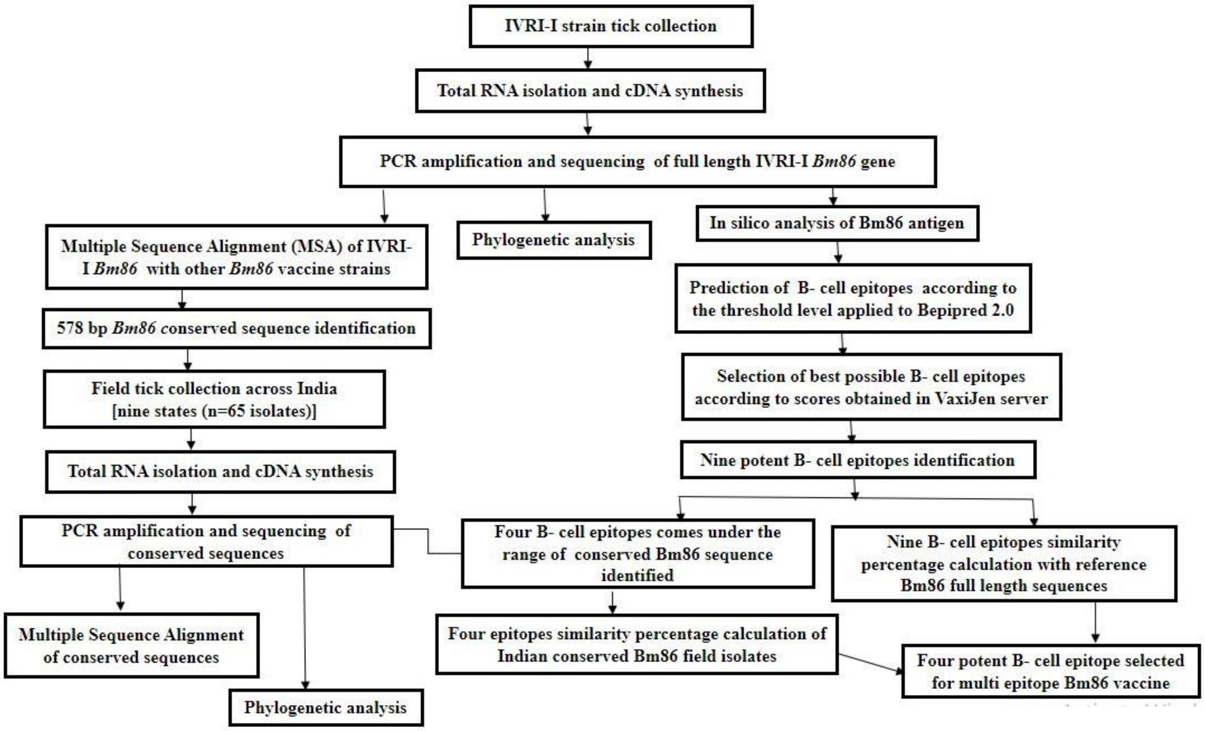

Figure S1: Flowchart of study methodology.

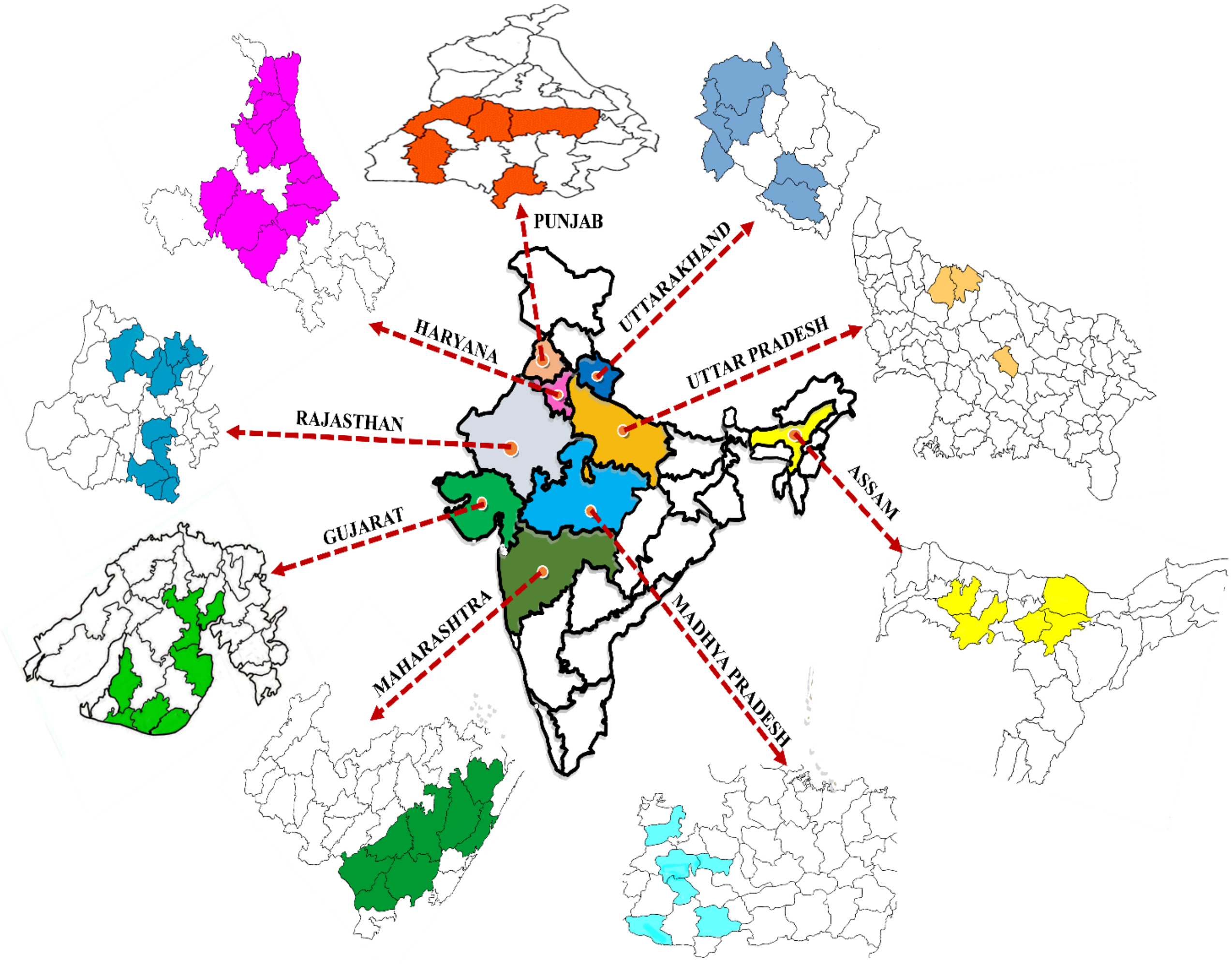

Figure S2: An outline map showing district wise sample collection sites.

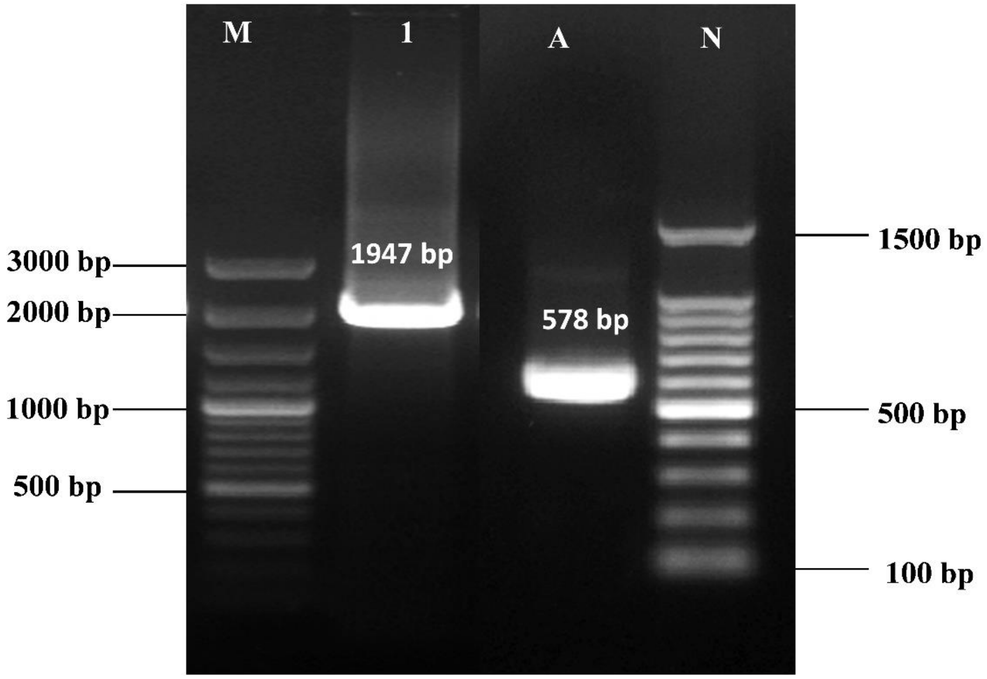

Figure S3: PCR amplification of full-length (1947 bp) and 578bp fragment of conserved *Bm86* gene sequence of IVRI-I (lane M: 100bp plus DNA ladder (Thermo Scientific, USA); lane 1:1947 bp PCR product; lane N: 100 bp DNA Ladder (Gold Biotechnology, USA); lane A: 578 bp PCR product).

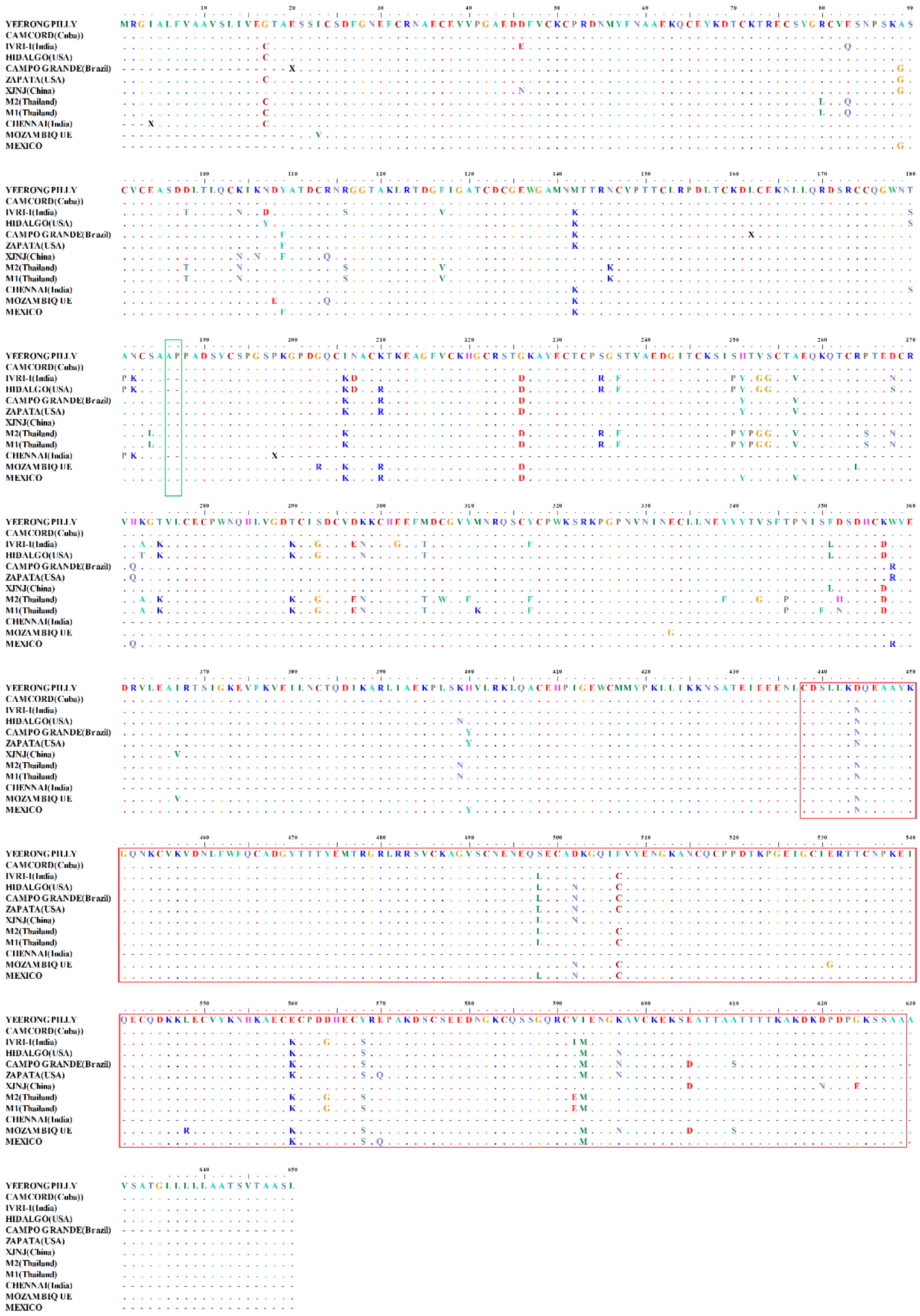

Figure S4: Multiple align sequence analysis of full-length IVRI-I *Bm86* gene with published vaccine strains; green box depicting the amino acid deletion in Indian *Bm86* sequence; red box showing the *Bm86* conservation sequence.

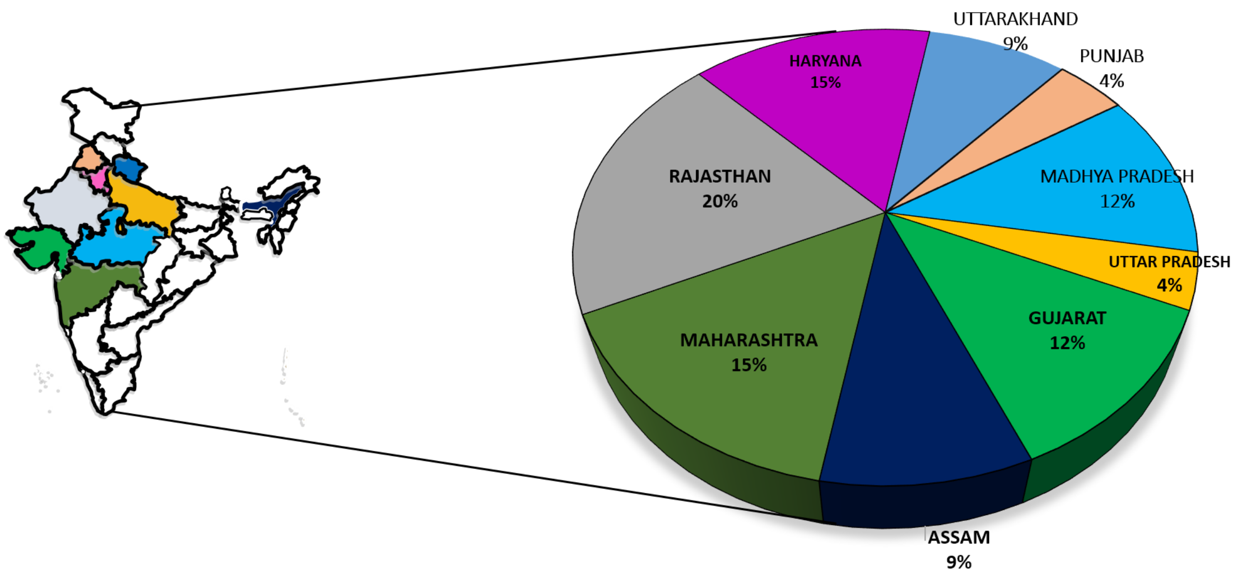

Figure S5: Pie chart representing the percentage of total amino acid share of each state (each state conserved Bm86 compared to IVRI-I *Bm86* conserved sequence).

Supplementary tables:

Table S1: Location of *Rhipicephalus microplus* engorged female tick samples collected across India.

State	District	Location a<br>Latitude, Longitude
1. Assam(N=6)	1. Barpeta	26.3216° N, 90.9821° E
	2.Dibrugarh	27.4728° N, 94.9120° E
	3.Kamrup	26.3161° N, 91.5984° E
	4.Sonitpur	26.6739° N, 92.8577° E
	5.Morigaon	26.2600° N, 92.2630° E
	6. Nagaon	26.3464° N, 92.6840° E
2. Rajasthan(N=12)	1.Alwara	27.5530° N, 76.6346° E
	2.Sikar	27.6094° N, 75.1399° E
	3.Jaipur	26.9124° N, 75.7873° E
	4.Udaipur	24.5854° N, 73.7125° E

	5.Chittorgarh	24.8887° N, 74.6269° E
	6.Pratapgarh	25.8973° N, 81.9453° E
	7.Bharatpur	27.2170° N, 77.4895° E
	8.Banswara	23.5461° N, 74.4350° E
	9.Bhilwara	25.3214° N, 74.5870° E
	10.Churu	28.2925° N, 74.9707° E
	11.Dausa	26.8932° N, 76.3375° E
	12.Dungarpur	23.8417° N, 73.7147° E
3. Maharashtra (N=9)	1.Jalgaon	21.0077° N, 75.5626° E
	2.Nashik	19.9975° N, 73.7898° E
	3.Dhule	20.9042° N, 74.7749° E
	4.Ahmednagar	19.0952° N, 74.7496° E
	5.Raigad	18.5158° N, 73.1822° E
	6.Pune	18.5204° N, 73.8567° E
	7.Aurangabad	19.8762° N, 75.3433° E
	8.Satara	17.6805° N, 74.0183° E
	9.Solapur	17.6599° N, 75.9064° E
4. Madhya Pradesh (N=6)	1.Mandsaur	24.0768° N, 75.0693° E
	2.Barwani	22.0363° N, 74.9033° E
	3. Shajapur	23.4273° N, 76.2730° E
	4.Ujjain	23.1793° N, 75.7849° E
	5.Indore	22.7196° N, 75.8577° E
	6.Khandwa	23.0853° N, 77.5791° E
5. Uttar Pradesh (N=4)	1.Lucknow	26.8467° N, 80.9462° E
	2. IVRI-I	28.4026° N, 79.4245° E
	3. Pilibhit	28.5835° N, 80.0088° E
	4. Raebareli	26.2145° N, 81.2528° E
6. Haryana(N=11)	1.Panipat	29.3909° N, 76.9635° E
	2.Kurukshetra	29.9695° N, 76.8783° E
	3.Yamuna Nagar	30.1290° N, 77.2674° E
	4.Kaithal	29.7857° N, 76.3985° E
	5.Ambala	30.3782° N, 76.7767° E
	6.Karnal	29.6857° N, 76.9905° E
	7.Hisar	29.1492° N, 75.7217° E
	8.Fatehabad	29.5077° N, 75.4520° E
	9. Bhiwani	28.7752° N, 75.9928° E
	10. Rohtak	28.8955° N, 76.6066° E
	11. Sonipat	29.0523° N, 76.9182° E
7. Uttarakhand (N=6)	1. Nainital	29.2794° N, 79.4704° E
	2.Haridwar	29.9457° N, 78.1642° E
	3.New Tehri	30.3739° N, 78.4325° E
	4.Uttarkashi	30.7268° N, 78.4354° E
	5.Dehradun	30.3165° N, 78.0322° E
	6. Almora	29.8150° N, 79.2902° E
8. Gujarat(N=7)	1.Ahmedabad	23.0225° N, 72.5714° E
	2.Junagadh	21.5222° N, 70.4579° E

	3.Porbandar	21.6417° N, 69.6293° E
	4.Jamnagar	22.4707° N, 70.0577° E
	5.Somnath	20.8882° N, 70.4060° E
	6.Bhavnagar	21.7645° N, 72.1519° E
	7.Anand	22.3299° N, 72.6151° E
9. Punjab(N=5)	1. Muktsar	30.4766° N, 74.5112° E
	2.Firozpur	30.9331° N, 74.6225° E
	3.Ludhiana	30.9010° N, 75.8573° E
	4.Mansa	28.8955° N, 76.6066° E
	5.Moga	30.8230° N, 75.1734° E

a These locations were based on https://maps.google.com

Table S2: Specific amino acid substitutions/mutations of full-length Indian (IVRI-I) *Bm86* gene with respect to commercial vaccine strains.

**1. IVRI-I amino acid mutations with respect to Yeerongpilly strain (TickGARD)**	**Percent of a.a changes/mutations**
G17C, D46E, E83Q, D98T,K104N,N107D, R116S, F127V, M142K, T180S, A181P, N182K, A186-, P187-, I206K, N207D, G226D, S235R, S237F, S250P, H251Y, V253G, S254G, A257V, D268N,K237A, T275K, T290K, S293G, D297E, K298N, E300G, M305T, Y317F, F351L,K357D, D444N,S498L,F507C, E560K, D564G, Y568S, V592I, I593M	44X100/650=**6.76%**
**2. IVRI-I amino acid mutations with respect to Camcord strain of Cuba (GAVAC)**<br>D27E, E64Q, D79T, K85N, N88D, I92T, R97S, F108V, M123K, T161S, A162P, N163K, A167-, P168-, I187K, N188D, G207D, S216R, S218F, S231P, H232Y,	44X100/609=**7.22%**

V234G, S235G, A238V, D249N, K254A, T256K, T271K, S274G, D278E, K279N, E283G, M286T, Y298F, F332L, K338D, D425N, S479L, F488C, E541K, D545G, Y549S, V573I, I574M	

Table S3: Specific amino acid substitutions/mutations in different Indian isolates conservation sequences with respect to Indian (IVRI-I) *Bm86* conservation sequence.

State	Isolates (total a. a changes)	Amino acid changes with respect to IVRI-I Bm86 conservation sequence
1. Assam	Nagaon (3)	N442D, I590V, K595N
	Barpeta (7)	N442D, A499T, D500N, G562D, H563R, I590V, K595N
	Kamrup (6)	N442D, D500N, G562D,E568Q, K595N,A614P
	Sonitpur(8)	N442D, A499T, D500N, G562Y, H563R, R567G, I590V, K595N
	Morigaon(5)	N442D, A499T, D500N, G562D, I590V
	Dibrugarh(6)	N442D, A499T, D500N, G562D, I590V, K595N
2. Rajasthan	Alwar(9)	N442D, A499T, D500N, E508K, G562D, H563R, R567G, I590V, K595N
	Sikar(8)	N442D, A499T, D500N, G562D, H563R, R567G, I590V, K595N

	Jaipur(8)	N442D, A499T, D500N, G562D, H563R, R567G, I590V,K595N
	Chittorgarh (9)	N442D, A499T, D500N, K521R, G562D, H563R, S566F, I590V,K595N
	Pratapgarh(5)	N442D, D500N, G562D, K595N, A614P
	Bharatpur(5)	N442D, D500N, G562D, K595N, A614P
	Banswara(5)	N442D, D500N, G562D, K595N, A614P
	Bhilwara(8)	N442D, A499T, D500N, D519G, G562D, H563R, I590V, K595N
	Churu(9)	N442D, A499T, D500N, G562D, H563R, R567G, I590V, N593D, K595N
	Dausa(10)	N442D, A499T, D500S, K521R, K554I, G562D, R567G, I590V, E603D, A614S
	Udaipur(10)	N442D, N459F, A499T, D500N, K86R, G562D, H563R, S566F, I590V, K595N
	Dungarpur(9)	N442D, L459F, A499T, D500N, D84G, K86R, G562D, S566F, K595N
3. Maharashtra	Jalgaon(10)	N442D, L459F, A499T, D500N, E89G, G562D, H563R, S566F, I590V, K595N
	Nashik(6)	N442D, D500N, G562D, E133Q, K595N, A614P
	Dhule(7)	N442D, A499T, D500N, G562D, I590V, K595N, A614P
	Ahmednagar(8)	N442D, A499T, D500N, E524G, G562D, E568Q, K595N, A614P
	Raigad(8)	N442D, C464R, D500N, G562D, H563R, R567G, I590V, K595N
	Pune(10)	N442D, A499T, D500N, G562D, H563R, S566F, I590V, K595N, K601T, D618N
	Aurangabad(8)	N442D, D500N, E508K, G562Y, H563R, R567G, I590V, K595N
	Satara(9)	N442D, A499T, D500N, G562Y, H563R, R567G, I590V, K601T, D616N
	Solapur(9)	N442D, A499T, D500N, G562Y, H563R, R567G, I590V, K601T, D616N
4. Madhya Pradesh	Khandwa(1)	N442D
	Shajapur(8)	N442D, A499T, D500N, G562D, H563R, R567G, I590V, K595N
	Barwani(9)	N442D, A499T, D500N, G562D, H563R, S566F, R567G, I590V, K595N
	Mandsaur(8)	N442D, A499T, D500N, G562D, H563R, R567G, I590V, K595N
	Indore(8)	N442D, A499T, D500N, G562D, H563R, R567G, I590V, K595N
	Ujjain(8)	N442D, A499T, D500N, G562D, H563R, R567G, I590V, K595N
5. Uttar Pradesh	Pilibhit (3)	N442D, D500N, D618N
	Raebareli(8)	N7D, D500N, G562D, H563R,S566F,I590V, K595N, E166T

	Lucknow(8)	N442D, D500N, G562D, H563R, S566F, I590V, K595N, E166T
6. Haryana	Panipat(9)	N442D, A499T, D500N, G562D, H563R, R567G, S575G, I590V, K595N
	Sonipat (10)	N442D, F460I, S482F, A499D, D500N, G562D, R567G, S575G, I590V, K595N
	Kurukshetra(8)	N442D, D500N, G562D, H563R, R567G, S575G, I590V, K595N
	Yamuna Nagar(1)	N442D
	Kaithal(3)	N442D, F460Y, G467D
	Ambala(11)	N442D, A499T, D500N, E508K, G562D, H563R, R567E, D578G, I590V, K595N, V597A
	Karnal(8)	N442D, A499T,D500N,G562D, H563R, R567G, I590V, K595N
	Hisar(8)	N442D, A499T, D500N, G562D, H563R, S566F, I590V, K595N
	Fatehabad(4)	N442D, T469K, E473G, A608S
	Bhiwani(7)	N442D, A499T, D500N, G562D, H563R, I590V, K595N
	Rohtak(3)	N442D, A499T, D500N
7. Uttarakhand	Mukteswar(9)	N442D, A499T, D500N, G562Y, H563R, R567G, E577V, I590V, K595N
	Haridwar(9)	N442D, A499T, D500N, G562Y, H563R, R567G, E577V, I590V, K595N
	New Tehri(10)	N442D, A499T, D500N, G562Y, H563R, R567G, E577V, I590V, K595N, K601T
	Uttarkashi(10)	N442D, A499T, D500N, E508K, G562Y, H563R, R567G, E577V, I590V, K595N
	Dehradun(9)	N442D, A499T, D500N, G562Y, H563R, R567G, E568Q, I590V, K595N
	Almora(10)	N442D, A499T, D500N, G562Y, H563R, R567G, E577V, I590V, K595N, K601T
8. Gujarat	Ahmedabad(9)	N442D, V483M, A499T, D500N, G562D, H563R, I590V, K595N, D616N
	Junagadh(10)	N442D, V483M, A499T, D500N, G562D, H563R, R567G, I590V, K595N, D616N
	Porbandar(9)	N442D, V483M, A499T, D500N, G562D, H563R, I590V, K595N, D616N
	Jamnagar(10)	N442D, V483M, A499T, D500N, K86R, G12D, H563R, I590V, K595N, D616N
	Somnath(9)	N442D, V483M, A499T, D500N, G562D, H563R, I590V, K595N,D616N
	Bhavnagar(10)	N442D, V483M, A499T, D500N, E508K, G562D, H563R, I590V, K595N, D616N
	Anand(9)	N442D, V483M, A499T, D500N. G562D, H563R, I590V, K595N, D616N
9. Punjab	Muktsar(4)	N442D, A499T, D500N, I590V
	Firozpur(4)	N442D, A499T, D500N, K595N

	Ludhiana(4)	N442D, A499T, D500N, H128D
	Mansa(4)	N442D, A499T, D500N, I590V
	Moga(5)	N442D, A499T, D500N, G562D

Table S4: The percentage similarity of conserved IVRI-I Bm86 B-cell epitopes with Indian *Bm86* conserved field isolates B-cell epitopes. The levels of amino acid similarity were classified as low (75–85%; green cells), medium (85–95%; yellow cells) and high (95–100%; red cells).

**Conserve Bm86 sequence B-cell epitope**	**1**	**2**	**3**	**4**	**5**	**6**	**7**	**8**	**9**
**Bm86(D519-K554)**	100%	100%	100%	100%	100%	100%	100%	100%	100%
**Bm86(H563-Q587)**	100%	96%	96%	96%	92%	100%	96%	88%	92%
**Bm86(C598-T606)**	100%	100%	100%	100%	100%	100%	100%	100%	91%
**Bm86(T609-K623)**	100%	100%	100%	100%	100%	100%	100%	100%	100%

1. Assam, 2. Gujarat. 3. Haryana, 4. Maharashtra, 5. Madhya Pradesh, 6. Punjab, 7. Rajasthan, 8. Uttarakhand, 9. Uttar Pradesh

Table S5: The impact of mutations on IVRI-I Bm86 B-cell epitope antigenicity with respect to the Bm86 vaccine strains (based on VaxiJen score analysis)

**B- cell epitope**	**VaxiJen scoresa**		
	**IVRI-I strain**	**Yeerongpilly strain**	**Camcord strain**
**Bm86(T18-D45)**	0.7950	0.7950	-
**Bm86(D97-G129)**	1.3361	1.4588	1.4588
**Bm86(G177-D224)**	0.9020	0.6583	0.6583
**Bm86(W280-R311)**	1.3679	1.1726	1.1726
**Bm86(K319-K501)**	0.6052	0.6446	0.6446
**Bm86(D519-K554)**	0.8751	0.8751	0.8751
**Bm86(H563-Q587)**	1.4514	1.4756	1.4756
**Bm86(C598-T606)**	1.4151	1.4151	1.4151
**Bm86(T609-K623)**	0.7273	0.7273	-

aThe VaxiJen score directly proportional to antigenicity of an epitope
